# ARNDT GOTTRON SCLEROMYXEDEMA: SUCCESSFUL RESPONSE TO TREATMENT WITH STEROID MINIPULSE AND METHOTREXATE

**DOI:** 10.4103/0019-5154.53183

**Published:** 2009

**Authors:** Vandana Mehta, C Balachandran, Raghavendra Rao

**Affiliations:** *From the Department of Skin and STD, Kasturba Hospital, Manipal - 576 104, Karnataka, India. E-mail: vandanamht@yahoo.com*

Sir,

Scleromyxedema is a rare disorder of unknown cause, with fewer than 150 reported cases in literature. It usually affects middle-aged adults of 30–50 years and is clinically characterized by a widespread symmetric eruption of 2–3 mm firm, waxy, dome-shaped papules, commonly over the hands, forearms, head and neck region, upper trunk, and thighs. The characteristic is the arrangement of papules in a striking linear array with the surrounding skin being sclerodermoid.[1] We report a case of scleromyxedema who considerably improved with a trial of steroid oral minipulse and methotrexate.

**Figure 1:**
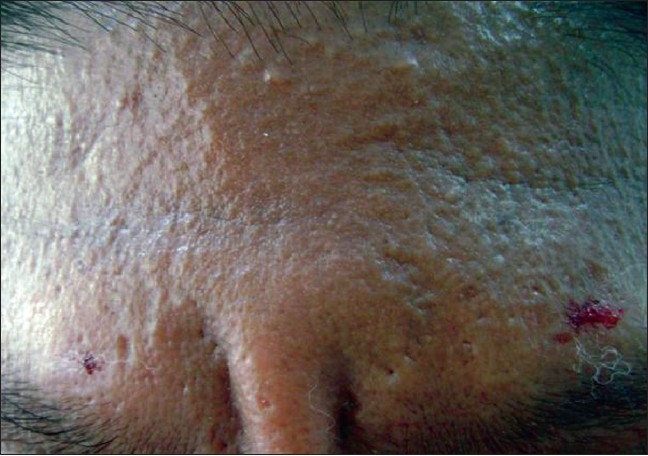
Infiltrated forehead skin with papules in a linear array

**Figure 2:**
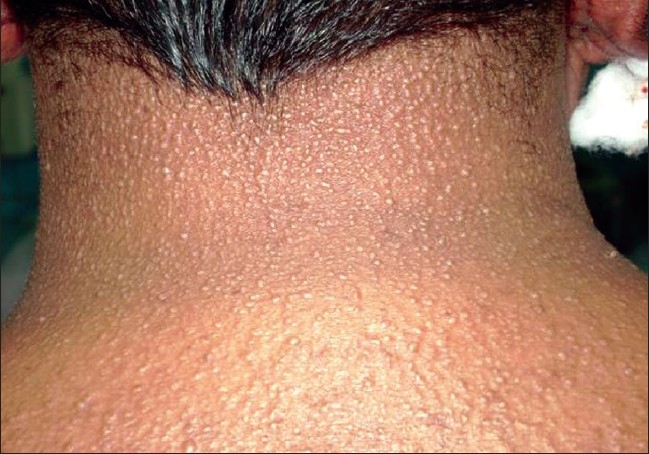
Skin-colored papules on the neck and back

A 43-year-old male farmer presented with a skin colored eruption on his body of 6 months duration. Initially, the lesions began as grouped skin-colored papules on his hands associated with mild pruritus and subsequently spread to the face, neck, shoulders, upper extremities, and the abdomen. There was no history of any systemic symptoms and his past medical and family history was noncontributory. On examination, facial and ear lobe infiltration was noted with prominent forehead creases. The skin of the forehead, neck, upper trunk, and arms was bound down and exhibited numerous 1–2 mm grouped skin colored papules [Figures 1 and 2]. Our patient was thoroughly investigated and results of his complete blood cell count, urinalysis, liver and renal parameters, thyroid profile, ECG, chest X-ray, ultrasound abdomen, and sugars were normal. ELISA for HIV was negative. Immunoglobulin profile showed elevated serum IgG 1797 (mg/dl) (normal 1200–1480 mg/dl), whereas IgA, IgM were normal. Biopsy features from the papule on the forearm showed features that are characteristic of scleromyxedema with special stains demonstrating mucin in the dermis.

In the literature, the terms lichen myxedematosus(LM), papular mucinosis, and scleromyxedema have often been used indiscriminately as synonyms, but most reported cases of LM without indication of the subtype appear infact to be cases of scleromyxedema. Actually, LM includes three distinct clinicopathologic subsets: A generalized papular and sclerodermoid form called as *scleromyxedema* with systemic even lethal manifestations, a localized papular form that does not run a disabling course and an atypical or intermediate form (not meeting the criteria for either scleromyxedema or the localised papular forms).[2] Patients of scleromyxedema may have a number of extracutaneous manifestations pertaining to the cardiovascular, pulmonary, gastrointestinal, rheumatologic, and central nervous systems. A paraproteinemia typically an IgGλ is observed in more than 80% patients. Microscopically, scleromyxedema is characterized by a triad of a diffuse deposit of mucin in the upper and mid reticular dermis, an increase in collagen deposition, and a marked proliferation of irregular arranged fibroblasts. The exact pathophysiologic mechanism that triggers excess mucin production by fibroblasts in scleromyxedema is unclear. Although this can be attributed to paraproteins according to previous studies, all patients of papular mucinosis do not have an identifiable paraprotein, which suggest the role of other cytokines as proliferative signals.[3]

The treatment of scleromyxedema remains a therapeutic challenge to the treating physician and despite anecdotal reports of success with various agents, no satisfactory treatments are currently available.[4]

Our patient was diagnosed as scleromyxedema clinically on the basis of a generalized papular and sclerodermoid eruption and histologically by the presence of mucin deposition fibrosis and fibroblast proliferation with a normal thyroid function. Extensive screening did not reveal any internal malignancy and although our patient had increased gamma globulin levels, we could not investigate this further due to financial constraints to support our diagnosis further. He was commenced on minipulse therapy with oral betamethasone six tablets to be taken twice weekly with weekly methotrexate 10 mgs. After 3 months of regular treatment, patient reported 75% reduction in the cutaneous induration. There were no other systemic complaints. Patient is currently on regular treatment and is doing well.
